# Anticariogenic Activity of Three Essential Oils from Brazilian Piperaceae

**DOI:** 10.3390/ph15080972

**Published:** 2022-08-06

**Authors:** Êni S. Carvalho, Vanessa F. S. Ayres, Midiã R. Oliveira, Geone M. Corrêa, Renata Takeara, Anderson C. Guimarães, Mariana B. Santiago, Thaís A. S. Oliveira, Carlos H. G. Martins, Antônio E. M. Crotti, Eliane O. Silva

**Affiliations:** 1 Departamento de Química Orgânica, Instituto de Química, Universidade Federal da Bahia, Salvador 40170-115, Brazil; 2 Instituto de Ciências Exatas e Tecnologia, Universidade Federal do Amazonas, Itacoatiara 69103-128, Brazil; 3 Instituto de Ciências Exatas, Universidade Federal do Amazonas, Manaus 69077-000, Brazil; 4 Instituto de Ciências Biomédicas, Universidade Federal de Uberlândia, Uberlândia 38405-318, Brazil; 5 Departamento de Química, Faculdade de Filosofia, Ciências e Letras de Ribeirão Preto, Universidade de São Paulo, Ribeirão Preto 14040-901, Brazil

**Keywords:** antibacterial activity, oral pathogens, *Piper callosum*, *Piper marginatum*, *Peperomia pellucida*

## Abstract

The current trend toward using natural food additives, cosmetics, and medicines has motivated industries to substitute synthetic compounds for natural products. Essential oils (EOs) from medicinal plants are a well-known source of chemical compounds that display several interesting biological activities, including antimicrobial action. In this study, we investigated the antibacterial activity of EOs extracted from three Piperaceae species collected in the Brazilian Amazon region against a representative panel of cariogenic bacteria. The minimum inhibitory concentration (MIC) of the essential oils extracted from *Peperomia pellucida* (PP-EO), *Piper marginatum* (PM-EO), and *Piper callosum* (PC-EO) was determined against *Streptococcus mutans*, *S. mitis*, *S. sanguinis*, *S. salivarius*, *S. sobrinus*, *Enterococcus faecalis*, and *Lactobacillus casei* by using the microplate microdilution method. PM-EO, PC-EO, and PP-EO displayed antibacterial activity against all the tested cariogenic bacteria. PM-EO displayed the best inhibitory activity, with MIC values ranging from 50 to 500 µg/mL. The lowest MIC values were obtained for PM-EO against *S. mitis* (MIC = 75 μg/mL), *Lactobacillus casei* (MIC = 50 μg/mL), and *S. mutans* (MIC = 50 μg/mL). Gas chromatography mass spectrometry (GC-MS) analysis allowed the chemical composition of all the EOs to be identified. The main constituents of PM-EO, PC-EO, and PP-EO were 3,4-(methylenedioxy)propiophenone, α-pinene, and dillapiole, respectively. Finally, the compounds that were exclusively detected in PM-EO are highlighted. Our results suggest that PM-EO may be used in products for treating dental caries and periodontal diseases.

## 1. Introduction

The current trend toward consuming minimally processed products has encouraged the development of alternative natural additives that can perform the same functions as synthetic additives. In this scenario, essential oils (EOs) extracted from medicinal plants have been screened for their safe and eco-friendly applications in the pharmaceutical, cosmetic, and food industries [1]. EOs are mixtures of volatile compounds produced by the specialized metabolism of plants to carry out survival functions [2]. Interesting biological properties, such as antimicrobial, antioxidant, anti-inflammatory, and anticancer activities, have been attributed to the complex chemical constitution of EOs [3]. Terpenoids, especially monoterpenes and sesquiterpenes, are the main chemical constituents of EOs and have been proven to play a key role in inhibiting pathogens [4]. Specifically, monoterpenoids affect microorganism multiplication and development by interfering with their physiological and biochemical processes [5].

Oral bacteria belonging to the genus *Streptococcus* can produce adhesive molecules that allow them to colonize different tissues in the mouth through biofilm formation [6]. The same bacteria can ferment carbohydrates, generating acids as by-products, which culminates in tooth demineralization and cavitation (dental caries) [7]. *Streptococcus mutans* is one of the main agents that cause dental caries: it colonizes the tooth surface and metabolizes different types of carbohydrates, using them to form biofilms on the tooth surface under low-pH conditions [8].

Because chlorhexidine exerts bacteriostatic and bactericidal effects against various microorganisms, it is the gold standard anticariogenic chemical agent [9]. However, synthetic oral biocides, including chlorhexidine, are falling into disuse due to the adverse effects associated with their frequent application and concerns about the emergence of microbial resistance to them [10]. Recently, our research group reviewed publications on the antibacterial activity of EOs extracted from several plants against bacteria that cause caries and periodontal diseases [11]. EOs are effective antimicrobials that can be employed as an alternative to traditional anticariogenic products, especially for long-term use.

Piperaceae, a large family of angiosperms, is composed of about 3700 species, with *Piper* and *Peperomia* being the most representative genera. In folk medicine, *Piper* and *Peperomia* species are used for treating many diseases, and numerous bioassays with essential oils, extracts, fractions, and pure compounds obtained from these plants have been reported [12,13]. Chemical analysis of EOs extracted from Piperaceae plants revealed the presence of monoterpenes, sesquiterpenes, and arylpropanoids [14] endowed with interesting biological properties. *Piper* species, also known as “pepper”, have well-known applications in gastronomy, and their secondary metabolites have a wide range of human health effects [15]. Of particular interest is the antimicrobial potential of the genus *Piper*, which has been useful for treating chronic periodontitis [16].

This study aimed to evaluate and compare the antimicrobial activity of the EOs extracted from fresh leaves of three Brazilian Piperaceae species (*Piper marginatum, Piper callosum*, and *Peperomia pellucida*) against a representative panel of cariogenic bacteria. The chemical composition of each EO was determined by GC-MS analysis.

## 2. Results

We obtained essential oils extracted from the leaves of *Piper marginatum* (PM-EO), *Piper callosum* (PC-EO), and *Peperomia pellucida* (PP-EO) as pale-yellow oils in 0.60%, 0.26%, and 0.04% yield (*w*/*w*), respectively. Table 1 shows the chemical constituents of PM-EO, PC-EO, and PP-EO, as identified by gas chromatography with flame ionization detection (GC-FID) and gas chromatography mass spectrometry (GC-MS) analysis. PC-EO, PM-EO, and PP-EO are rich in phenylpropanoids (31.6%, 32.4%, and 41.7%, respectively), but they differ in content of monoterpenes (18.6%, 48.3%, and 0.7%, respectively) and sesquiterpenes (44.8%, 18.8%, and 52.4%, respectively). Monoterpene hydrocarbons (44.9%) and oxygenated sesquiterpenes (35.2%) predominate in PC-EO, whereas PM-EO is rich in monoterpene and sesquiterpene hydrocarbons (18.6% and 36.0%, respectively). The major compounds identified in PM-EO, PC-EO, and PP-EO were 3,4-(methylenedioxy)propiophenone (11.3%), α-pinene (19.2%), and dillapiole (40.6%), respectively. 3,4-(methylenedioxy)propiophenone (11.3%), myristicin (5.3%), croweacin (5.2%), δ-3-carene (4.6%), and (*Z*)-β-ocimene (4.2%), which are relatively abundant in PM-EO, were not detected in PC-EO or PP-EO.

We assessed the in vitro antibacterial activities of PM-EO, PC-EO, and PP-EO by using the microplate microdilution method and evaluated them in terms of the minimum inhibitory concentration (MIC, i.e., the lowest concentration of the compound capable of inhibiting the growth of cariogenic bacteria). We performed the assay against seven bacteria (*Streptococcus mutans*, *S. mitis*, *S. salivarius*, *S. sanguinis*, *S.* *sobrinus, Enterococcus faecalis*, and *Lactobacillus casei*) and used chlorhexidine dihydrochloride as the positive control. DMSO was used to solubilize the essential oils and assayed as the negative control. No inhibitory effect of DMSO on the bacteria was observed. The results are depicted in Table 2.

The MIC values obtained for PM-EO, PC-EO, and PP-EO were in the ranges of 50–500, 500–1000, and 125–1000 μg/mL, respectively. These EOs were less effective against *E. faecalis* (MIC values from 500 to 1000 µg/mL) and *S. sanguinis* (MIC values from 225 to 1000 µg/mL). On the other hand, PM-EO, PC-EO, and PP-EO were more effective against *S. mutans* and *L. casei* (MIC values from 50 to 500 µg/mL). The lowest MIC values were achieved with PM-EO against *S. mutans* (MIC = 50 μg/mL), *L. casei* (MIC = 50 μg/mL), and *S. mitis* (MIC = 75 μg/mL).

## 3. Discussion

A comparison between the chemical composition of the essential oils extracted from *Piper marginatum* (PM-EO), *P. callosum* (PC-EO), and *Peperomia pellucida* (PP-EO) revealed that, although these EOs are rich in phenylpropanoids, the chemical profiles of the EOs obtained from the two *Piper* species (PM-EO and PC-EO) differ from that of PP-EO in terms of the contents of monoterpene hydrocarbons (which is lower in PP-EO) and oxygenated sesquiterpenes (which is higher in PP-EO). PM-EO and PC-EO are rich in monoterpene and sesquiterpene hydrocarbons; however, PC-EO is rich in monoterpene hydrocarbons, whereas sesquiterpene hydrocarbons are richest in PP-EO.

The main constituents of PC-EO are α-pinene (19.2%), β-pinene (14.3%), and methyl eugenol (6.5%). Interestingly, PC-EO is rich in α- and β-pinene, whereas safrole has frequently been highlighted as the main compound in EOs extracted from *P. callosum* [33,34,35].

PP-EO contains dillapiole (40.6%), (*E*)-caryophyllene (13.2%), viridiflorol (15.1%), and bicyclogermacrene (9.1%) as the main compounds. Phenylpropanoids were reported as the major compounds in the EOs obtained from *P. pellucida* [36]; the most predominant chemotype was shown to contain dillapiole as the main compound [37]. The chemical composition of *P. pellucida* specimens collected in the Brazilian Amazon, Rio de Janeiro (Brazil), and Cameroon are marked by the presence of dillapiole [28,38,39,40].

Here, we identified 3,4-(methylenedioxy)propiophenone (11.3%), germacrene-D (10.8%), and *E*-β-ocimene (7.7%) as the major compounds in PM-EO. Da Silva et al. reported 3,4-methylenedioxypropiophenone (21.8%) as the main component in the EO extracted from another *P. marginatum* specimen collected in the Brazilian Amazon region [33]. In addition, 3,4-methylenedioxypropiophenone (22.9%), δ-3-carene (10.2%), (*E*)-caryophyllene (9.7%), and spathulenol (6.9%) were detected as the main components in the EO extracted from *P. marginatum* leaves collected in Santarém, Pará, Brazil [41]. On the other hand, the EOs extracted from the leaves of a *P. marginatum* specimen collected in the State of Pernambuco, northeastern Brazil, were shown to contain (*Z*)- or (*E*)-asarone (30.4 and 6.4%, respectively) and patchouli alcohol (16.0%) as the main compounds [42]. The EO extracted from *P. marginatum* collected in Curitiba, Paraná, Brazil, was reported to contain myristicin (12.8%), sarisan (12.3%), and kakuol (13.3%) as the main compounds [43].

Differences exist in the quantitative and qualitative profiles of EOs extracted from specimens collected worldwide, and are associated with environmental factors or growing conditions, which greatly affect the chemical composition of volatile oils and, hence, their biological activities [44].

The use of plant species as sources of alternative therapeutic agents for infectious diseases is noteworthy. Mouthwashes containing EOs provided promising results in terms of inhibition of pathogenic oral microorganisms [45] through several mechanisms, such as cell wall disruption, inhibition of enzymatic activity, and biofilm formation [46,47]. Ethanol extracts from *Piper* species inhibit the bacteria that cause oral diseases [16]. Antimicrobial assays showed that the EO extracted from *Piper muricatum* Blume (Piperaceae) has moderate activity toward *Bacillus cereus* and *Streptococcus mutans* (MIC values of 250 µg/mL) [48]. However, to the best of our knowledge, the antibacterial activity of EOs extracted from *Piper marginatum* (PM-EO), *Piper callosum* (PC-EO), and *Peperomia pellucida* (PP-EO) against cariogenic bacteria has not been reported.

Currently, the antibacterial activity of EOs against oral pathogens can be classified based on their MIC values. According to Oliveira et al., EOs with MIC values lower than 100 µg/mL, between 101 and 500 µg/mL, between 501 and 1500 µg/mL, and between 1500 and 2000 µg/mL are considered very active, active, moderately active, and weakly active, respectively. MIC values higher than 2000 µg/mL denote an inactive EO [11]. According to these criteria, PM-EO, PC-EO, and PP-EO display antibacterial activity against all the tested cariogenic bacteria. PC-EO displays moderate activity, with MIC values ranging from 500 µg/mL (against *S. mutans*, *S. mitis*, *S. salivarius*, *S. sobrinus*, and *L. casei*) to 100 µg/mL (against *S. sanguinis* and *E. faecalis*). On the other hand, PM-EO is the most active among the assayed EOs. The very strong activity of PM-EO against *S. mutans* (MIC = 50 µg/mL) is noteworthy: this bacterium is one of the main microorganisms underlying caries because it can produce both soluble and insoluble glucans from dietary sucrose by using glucosyltransferases [49]. Natural products with antimicrobial effects are an attractive alternative to conventional synthetic agents for preventing dental caries [10].

Studies on the antibacterial activity of EOs have been carried out because such oils do not elicit bacterial resistance given that they are mixtures of compounds [11]. Lipophilic constituents of EOs successfully inhibit microbial growth because they react with the lipid parts of cell membranes; in addition, they inhibit the synthesis of DNA, RNA, proteins, and polysaccharides in bacterial cells [50]. Of the three Piperaceae EOs we evaluated herein, PM-EO has the most promising antibacterial activity. GC-MS analysis of its chemical constituents showed a great amount of 3,4-(methylenedioxy)propiophenone (11.3%), myristicin (5.3%), croweacin (5.2%), δ-3-carene (4.6%), and (*Z*)-β-ocimene (4.2%), which we did not detect in PC-EO or PP-EO. The biological activities displayed by EOs are due to their chemical composition and may originate from the action of a specific compound or the synergistic action of all the chemical compounds in the EO [51]. In this study, we surveyed the literature to determine the antimicrobial activities displayed by the constituents exclusively detected in PM-EO, which could explain its stronger inhibitory activity on cariogenic bacteria.

Over the last few years, studies conducted with myristicin, one of the major compounds in PM-EO, have demonstrated its promising biological activities [52]. EOs containing myristicin as the main component have been shown to display interesting antimicrobial activities in food systems [53]. The EOs extracted from dill (*Anethum graveolens*) and parsley (*Petroselinum crispum*) grown during the summer and winter contain from 28% to 42% myristicin and was shown to inhibit *Escherichia coli*, *Staphylococcus albus*, *Bacillus mesentericus*, and *Aspergillus flavus* [54]. The EO extracted from *Pycnocycla bashagardiana* aerial parts contains 39% myristicin and was reported to exhibit strong antimicrobial activity against *Staphylococcus aureus*, *Staphylococcus epidermidis*, *Escherichia coli*, and *Candida albicans* [55]. Myristicin isolated from the EO of *Piper sarmentosum* (representing about 81% to 83% of its composition) was shown to inhibit the proliferation of *Escherichia coli* and the in vitro activity of the GTPase enzyme, interfering with a fundamental step for microbial cell division [56]. Apart from the antibacterial activities reported for myrsticin, δ-3-carene and (*Z*)-β-ocimene, which are major compounds in PM-EO, were also correlated with antimicrobial activities [57,58].

Despite the reported application of EOs in the pharmaceutical, cosmetic, sanitary, and food industries, recent studies demonstrated that EOs can exert prooxidant and cytotoxic effects on eukaryotic cells. Studies showed that EOs display cytotoxic effects, and their cytotoxic mechanisms were identified by examining gene and protein expression levels [59]. Depending on type and concentration, EOs can exhibit cytotoxic effects on living cells, even at low concentrations (IC_50_ 27.81 µg/mL) [60]. Therefore, the effective use of PM-EO, PC-EO, and PP-EO in oral formulations requires carefully evaluating their cytotoxicity. Complementing such discussion, PM-EO displayed the most promising anticariogenic activity, and its chemical analysis showed high content of sesquiterpene hydrocarbon. Comparisons between the toxicities of EOs distinguished by their content of sesquiterpene hydrocarbon and oxygenated sesquiterpenes were reported. An EO rich in sesquiterpene hydrocarbon displayed selective action, i.e., it was more toxic against cancer than noncancerous cells [61].

## 4. Materials and Methods

### 4.1. Plant Material

Leaves from *Piper marginatum*, *Piper callosum*, and *Peperomia pellucida* (Piperaceae) were all collected near the city of Itacoatiara, State of Amazonas, Brazil (S 03°01′50.5″–W 58°32′37.3″, S 03°04′28.6″–W 58°28′36.3″, and S 03° 08′ 28.8″–W 58° 26′ 54.3″, respectively) in March 2019 and identified by Prof. Dr. Ari de Freitas Hidalgo. Voucher specimens (8266, 8267, and 8264, respectively) were deposited at the Herbarium of the Federal University of Amazonas. This study was registered in the Brazilian System for the Management of Genetic Heritage and Associated Traditional Knowledge (SisGen) under codes AF36A53 and A2CE4A6.

### 4.2. Essential Oil Extraction

Fresh leaves (1200 g) of each species were divided into three samples (400 g each) and accommodated in 1 L round-bottom flasks containing 500 mL of distilled water. The flasks containing the fresh leaves were connected to a Clevenger-type apparatus and submitted to hydro-distillation for 3 h. After manual collection of the EOs, the obtained volume was measured, and traces of water were removed by freezing the sample below 0 °C, followed by transfer of the unfrozen EO to a new vial. The EO yields (*w*/*w*) were calculated from the weight of the fresh leaves. The EOs were conditioned in hermetically sealed glass containers at −20 °C until use.

### 4.3. Identification of the EO Compounds

The EOs were dissolved in ethyl ether and analyzed on a Shimadzu GC2010 Plus gas chromatograph (Shimadzu Corporation, Kyoto, Japan) equipped with an AOC-20s autosampler and fitted with FID and a data-handling processor. An Rtx-5 (Restek Co., Bellefonte, PA, USA) fused silica capillary column (30 m × 0.25 mm i.d.; 0.25 μm film thickness) was employed. The operation conditions were as follows: the column temperature was programmed to rise from 60 to 240 °C at 3 °C/min, then held at 240 °C for 5 min; the carrier gas was helium (99.999%) at a flow rate of 1.0 mL/min; injection mode; injection volume of 0.1 μL (split ratio of 1:10); injector and detector temperatures of 240 and 280 °C, respectively. The relative concentrations of the components were obtained by peak area normalization (%). The relative areas were the average of triplicate GC-FID analyses.

The GC-MS analyses were carried out on a Shimadzu QP2010 Plus (Shimadzu Corporation, Kyoto, Japan) system equipped with an AOC-20i autosampler. The column consisted of an Rtx-5MS (Restek Co., Bellefonte, PA, USA) fused silica capillary (30 m length × 0.25 mm i.d. × 0.25 μm film thickness). Electron ionization mode was used at 70 eV. Helium (99.999%) was employed as the carrier gas at a constant flow of 1.0 mL/min. The injection volume was 0.1 μL (split ratio of 1:10). The injector and the ion-source temperatures were set at 240 and 280 °C, respectively. The oven temperature program was the same as the one used for GC. The mass spectra were taken with a scan interval of 0.5 s for mass ranging from 40 to 600 Da. The DA-EO components were identified based on their retention indices on an Rtx-5MS capillary column under the same operating conditions used for GC, relative to a homologous series of n-alkanes (C8–C20) [62]. Structures were computer-matched to the Wiley 7, NIST 08, and FFNSC 1.2 spectral libraries, and their fragmentation patterns were compared to the literature data.

### 4.4. Bacterial Strains and Antimicrobial Assays

The minimum inhibitory concentration (MIC) values of the EOs were calculated by using the broth microdilution method in 96-well microplates. The following standard strains were employed: *Enterococcus faecalis* (ATCC 4082), *Streptococcus salivarius* (ATCC 25975), *Streptococcus sobrinus* (ATCC 33478), *Streptococcus mutans* (ATCC 25175), *Streptococcus mitis* (ATCC 49456), *Streptococcus sanguinis* (ATCC 10556), and *Lactobacillus casei* (ATCC 11578). Individual 24 h colonies from blood agar (Difco Labs, Detroit, MI, USA) were suspended in 10.0 mL of tryptic soy broth (Difco, Detroit, USA). The standardization of each microorganism suspension was carried out as previously described [50]. The EO samples were dissolved in DMSO (Merck, Darmstadt, Germany) at 1 mg/mL and diluted in tryptic soy broth (Difco) so that concentrations in the range from 4000 to 3.9 µg/mL would be achieved. The final DMSO concentration was 5% (*v*/*v*), and this solution was used as the negative control. One inoculated well was included to control the adequacy of the broth for organism growth. One noninoculated well free of the antimicrobial agent was also included to ensure medium sterility. Chlorhexidine dihydrochloride (C8527 Sigma) was dissolved in tryptic soy broth (Difco) and used as the positive control at concentrations ranging from 59.0 to 0.115 µg/mL. The microplates (96-well) were sealed with plastic film and incubated at 37 °C for 24 h. Next, 30 µL of 0.02% resazurin (199303 Sigma, St. Louis, MO, USA) aqueous solution was poured into each microplate reservoir to indicate microorganism viability. Visual readings of the resazurin color changing from blue (no bacterial growth) to pink (bacterial growth) were carried out. The MIC values were determined as the lowest concentration of each EO capable of inhibiting microorganism growth. Three replicate assays were accomplished for each microorganism.

## 5. Conclusions

The EOs extracted from fresh leaves of Brazilian populations of Piperaceae have promising activity against cariogenic bacteria. The main constituents detected in the samples evaluated in our study were 3,4-(methylenedioxy)propiophenone for PM-EO, α-pinene for PC-EO, and dillapiole for PP-EO. As for the chemical compounds detected in the EOs obtained from plants collected in other countries, we noted that 3,4-(methylenedioxy)propiophenone and dillapiole have frequently been identified as the main compounds of EOs obtained from *P. marginatum* and *P. pellucida*, respectively. On the other hand, safrole has been the main compound detected in the EO extracted from *P. callosum*. Some chemical compounds, such as myristicin, were exclusively detected in PM-EO and deserve attention because it displayed the most promising inhibitory activities against the evaluated oral bacteria during the MIC assays. The results presented herein suggest the possible use of Brazilian Piperaceae EOs in oral health products for treating dental caries and periodontal diseases, which emphasize their great potential for commercial application in phytomedicines. Our results provide new insights for the continuity of the evaluation of PM-EO, PC-EO, and PP-EO as oral products. Further cytotoxicity assays are necessary to reinforce the safety of their use in pharmaceuticals.

## Figures and Tables

**Table 1:** Chemical compounds detected in the EOs extracted from *Piper callosum* (PC-EO), *Piper marginatum* (PM-EO), and *Peperomia pellucida* (PP-EO). The compounds were quantified by GC-FID and identified by EI-MS.

Compound	RI_exp_	RI_lit_	% RA PM-EO	% RA PC-EO	% RA PP-EO	Identification
α-thujene	924	931	-	0.2 ± 0.09	-	RL ^a^ MS
α-pinene	932	939	0.9 ± 0.09	19.2 ± 0.88	-	RL ^a^ MS
camphene	948	953	-	0.6 ± 0.27	-	RL ^a^ MS
sabinene	971	976	-	2.7 ± 0.92	-	RL ^a^ MS
β-pinene	978	980	0.6 ± 0.05	14.3 ± 0.64	-	RL ^a^ MS
myrcene	988	991	0.6 ± 0.07	0.9 ± 0.37	-	RL^a^ MS
α-phellandrene	1007	1005	-	0.2 ± 0.08	-	RL ^a^ MS
δ-3-carene	1009	1011	4.6 ± 0.50	-	-	RL ^b^ MS
α-terpinene	1016	1018	-	1.4 ± 0.57	-	RL ^a^ MS
p-cymene	1024	1026	-	0.3 ± 0.09	-	RL ^a^ MS
limonene	1028	1031	-	0.8 ± 0.32	0.2 ± 0.03	RL ^a^ MS
1,8-cineole	1031	1033	-	2.3 ± 0.73	-	RL ^a^ MS
*Z*-β-ocimene	1038	1040	4.2 ± 0.42	-	-	RL ^a^ MS
*E*-β-ocimene	1047	1050	7.7 ± 0.84	-	0.5 ± 0.09	RL ^a^ MS
γ-terpinene	1058	1062	-	3.5 ± 0.46	-	RL ^a^ MS
α-terpinolene	1084	1088	-	0.8 ± 0.27	-	RL ^a^ MS
linalool	1101	1098	-	0.1 ± 0.02	-	RL ^a^ MS
terpinen-4-ol	1180	1179	-	0.6 ± 0.17	-	RL ^a^ MS
hexyl butanoate	1192	1191	-	-	0.1 ± 0.04	RL ^c^ MS
α-terpineol	1195	1197	-	0.4 ± 0.02	-	RL ^d^ MS
decanal	1207	1207	-	-	1.3 ± 0.13	RL ^e^ MS
safrole	1290	1285	-	2.3 ± 0.03	-	RL ^c^ MS
δ-elemene	1332	1340	2.1 ± 0.17	-	-	RL ^a^ MS
α-copaene	1371	1376	-	1.2 ± 0.28	-	RL ^a^ MS
β -bourbonene	1379	1355	-	-	0.3 ± 0.06	RL ^a^ MS
β-elemene	1393	1391	0.7 ± 0.05	-	0.5 ± 0.05	RL ^a^ MS
methyl eugenol	1400	1403	0.7 ± 0.06	6.5 ± 1.13	-	RL ^a^ MS
dodecanal	1411	1409	-	-	0.6 ± 0.03	RL ^f^ MS
*E*-caryophyllene	1417	1418	5.5 ± 0.39	1.5 ± 0.34	13.2 ± 0.28	RL ^a^ MS
*trans*-α-bergamotene	1433	1438	-	-	0.1 ± 0.03	RL ^g^ MS
croweacin	1450	1452	5.2 ± 0.27	-	-	RL ^h^ MS
α-humulene	1452	1454	0.7 ± 0.06	0.4 ± 0.09	0.8 ± 0.31	RL ^a^ MS
*E*-β-farnesene	1457	1458	-	-	0.5 ± 0.02	RL ^a^ MS
γ-gurjenene	1469	1473	-	-	2.9 ± 0.70	RL ^i^ MS
α-amorphene	1471	1485	0.7 ± 0.06	-	-	RL ^a^ MS
germacrene-D	1475	1480	10.8 ± 0.73	2.6 ± 0.63	6.8 ± 0.77	RL ^a^ MS
β-selinene	1484	1485	2.4 ± 0.17	-	-	RL ^i^ MS
bicyclogermacrene	1491	1494	1.0 ± 0.25	-	9.1 ± 0.26	RL ^a^ MS
α-muurolene	1493	1499	1.4 ± 0.77	0.2 ± 0.04	-	RL ^a^ MS
*Z-*methyl isoeugenol	1496	1532	0.6 ± 0.08	-	-	RL ^a^ MS
germacrene A	1502	1503	0.7 ± 0.09	-	0.1 ± 0.06	RL ^a^ MS
γ-cadinene	1512	1513	-	-	0.1 ± 0.05	RL ^a^ MS
myristicin	1516	1520	5.3 ± 0.23	-	-	RL ^a^ MS
δ-cadinene	1523	1524	-	0.9 ± 0.05	-	RL ^a^ MS
β-sesquiphellandrene	1528	1524	-	-	0.8 ± 0.05	RL ^j^ MS
elemicin	1542	1540	9.2 ± 0.76	3.1 ± 0.70	-	RL ^l^ MS
3,4-(methylenedioxy)propiophenone	1543	1545	11.3 ± 0.03	-	-	RL ^m^ MS
*E*-nerolidol	1564	1564	-	-	1.3 ± 0.77	RL ^a^ MS
spathulenol	1572	1576	1.6 ± 0.07	-	0.4 ± 0.08	RL ^a^ MS
caryophyllene oxide	1578	1581	-	-	0.4 ± 0.07	RL ^a^ MS
globulol	1581	1584	0.6 ± 0.05	-	-	RL ^n^ MS
viridiflorol	1588	1590	0.6 ± 0.06	-	15.1 ± 0.32	RL ^i^ MS
10-*epi*-γ-eudesmol	1618	1621	0.7 ± 0.05	-	-	RL ^o^ MS
dillapiole	1620	1622	-	-	40.6 ± 0.90	RL ^i^ MS
γ-eudesmol	1629	1630	-	2.5 ± 0.77	-	RL ^i^ MS
isospathulenol	1635	1639	0.9 ± 0.09	-	-	RL ^p^ MS
torreyol	1642	1645	0.6 ± 0.05	1.0 ± 0.17	-	RL ^q^ MS
β-eudesmol	1650	1649	4.5 ± 0.03	-	-	RL ^i^ MS
apiole	1681	1680	-	-	1.1 ± 0.08	RL ^i^ MS
Monoterpene hydrocarbons			18.6	44.9	0.7	
Oxygenated monoterpenes			-	3.4	-	
Sesquiterpene hydrocarbons			36.0	13.3	35.2	
Oxygenated sesquiterpenes			8.8	3.5	17.2	
Phenylpropanoids			31.6	32.4	41.7	
Others			-	-	2.0	
Not identified			5.0	2.5	3.2	

RI: retention indices relative to *n*-alkanes C8–C20 on Rtx-5MS capillary column; RA: relative area* (peak area relative to the total peak area in the GC-FID chromatogram); RL: comparison of the retention index with the literature (a–q: [17,18,19,20,21,22,23,24,25,26,27,28,29,30,31,32], respectively); MS: comparison of the mass spectrum with the literature. * Average from three replicates.

**Table 2:** In vitro antibacterial activity (MIC; μg/mL) of the essential oils extracted from *Piper marginatum* (PM-EO), *Piper callosum* (PC-EO), and *Peperomia pellucida* (PP-EO) against cariogenic bacteria.

Microorganism	PC-EO	PM-EO	PP-EO	CHD
*Streptococcus salivarius* ATCC 25975	500	200	500	0.74
*Streptococcus sanguinis* ATCC 1055	1000	225	250	0.74
*Streptococcus sobrinu*s ATCC 33478	500	200	250	0.18
*Streptococcus mitis* ATCC 49456	500	75	125	1.47
*Streptococcus mutans* ATCC 25175	500	50	125	0.09
*Enterococcus faecalis* ATCC 4082	1000	500	1000	2.95
*Lactobacillus casei* ATCC 11578	500	50	125	0.37

CHD: chlorhexidine dihydrochloride (μg/mL), positive control.

## Data Availability

Data is contained within the article.
